# Recent Advances in Pancreatic Neoplasms

**DOI:** 10.3390/jcm10204724

**Published:** 2021-10-14

**Authors:** Cosimo Sperti, Simone Serafini, Lucia Moletta

**Affiliations:** Department of Surgery, Oncology and Gastroenterology, 3rd Surgery Clinic, University of Padua, Via Giustiniani 2, 35128 Padua, Italy; simone.serafini@ymail.com (S.S.); lucia.moletta@unipd.it (L.M.)

Pancreatic neoplasms, both primary and secondary, include different pathological entities with variable biological behavior and, consequently, different treatment modalities. Pancreatic adenocarcinoma is the fourth cause of cancer-related death in Western countries, and, in the coming years, it is estimated to become the second cause of gastrointestinal cancer death [1]. Surgery and adjuvant therapy are the cornerstones of the therapeutic approach; however, even after radical resection, the majority of patients experience disease recurrence. A multimodal therapeutic approach based on the combination of neoadjuvant therapy, chemotherapy, radiotherapy, immunotherapy, and surgery, appears fundamental in order to improve the outcomes, but the prognosis of pancreatic cancer remains dismal [2]. This Special Issue of the *Journal of Clinical Medicine*, entitled “Recent Advances in Pancreatic Neoplasms”, focuses on new possible strategies to treat pancreatic neoplasms, especially pancreatic adenocarcinoma (PDCA). This Special Issue contains 13 articles, 11 studies and 2 review articles, focusing on the pancreatic ductal adenocarcinoma (*n* = 10), intraductal papillary mucinous neoplasm (IPMN) (*n* = 1), neuroendocrine tumors (*n* = 1) and secondary tumor to the pancreas (*n* = 1) [3,4,5,6,7,8,9,10,11,12,13,14,15]. Seven papers [4,5,6,9,10,11,14] explored new prognostic factors potentially able to stratify patients with pancreatic cancer and different survival rates in order to select the adequate treatment. Allen et al. [6] examined the relationship between the daily rate of change in CA 19-9 over the first 90 days of chemotherapy for unresectable and metastatic pancreatic cancer and the pretreatment levels of neutrophils, lymphocytes and platelets with an overall progression-free survival. They found that the ratio of absolute neutrophils count to the absolute platelet count (NLR) was associated with a shorter overall survival (OS) and progression-free survival (PFS). As in other tumors, NLR could be considered a prognostic marker.

Gablo et al. [9] from the Czech Republic, studied miRNAs as prognostic biomarkers in the preoperative blood plasma specimen of patients with PDCA. A population of patients with a poor prognosis (OS < 16 months) and patients with a good prognosis (>20 months) were considered in the study. Two miRNAs were confirmed to have lower levels and one miRNA was confirmed to have higher levels in the plasma sample of poor prognosis cases. By combining these three miRNA levels, poor prognosis cases were identified with a sensitivity of 85% and a specificity of 80%.

In a multicentric retrospective study, Catalano et al. [5] evaluated the correlation between the occurrence of treatment-related peripheral neuropathy and the efficacy of the nab-P/Gem combination in patients with metastatic pancreatic adenocarcinoma. Peripheral neuropathy was the most frequent treatment-related adverse event and, in this study, patients developing peripheral neuropathy seemed to experience more favorable outcomes compared to patients without neuropathy.

Li et al. [13] from Madrid, Spain, evaluated the expression of several proteins (PIWIL1, PIWIL2, PIWIL3, PIWIL4) in pancreatic cancer-derived cell lines and in healthy control in one non-tumor cell line. These proteins played a role in regulating gene expression through the complementary recognition and guidance of short RNAs against their target genes. They also played a role in recognizing and binding a specific type of non-coding small RNAs: the piRNAs (PIWI-interacting RNAs), constituting the piRNA-induced silencing complex (piRISC). PIWI proteins are fundamental for epigenetic regulation, silencing transposable elements, protecting genome integrity, gametogenesis and piRNA biogenesis. The authors investigated the PIWI protein functions and their controversial role in tumorigenesis. According to this paper, PIWIL3 and PIWIL4 seemed to have a crucial role in the regulation of cell motility, stem cell maintenance and drug resistance both in tumorous and healthy pancreatic cells. Furthermore, a low PIWIL4 expression predicted a shorter survival time for patients with a pancreatic carcinoma.

The tumor marker CA 19-9 was proven to be useful in the clinical management of patients with PDAC, especially in monitoring the effects of treatment. Lee et al. [11] evaluated the association of CA 19-9 concentrations after neoadjuvant therapy (NACT) and the prognosis in a large number of patients with border-line resectable or locally advanced PDCA who underwent subsequent surgery. The authors considered the CA 19-9 concentrations before and after NACT, and after surgery, and calculated the relative difference (RDC) as follows: [(CA19-9 after NACT) − (CA 19-9 before NACT)]/(CA 19-9 before NACT). By constructing prognostic models for the overall survival and recurrence of free survival, the authors found RDC to be independently associated with a better prognosis in patients with border-line resectable or locally advanced PDCA.

Sperti et al. [10] from the University of Padua (Italy) retrospectively evaluated the prognostic implication of 18-FDG PET in resectable pancreatic cancer. One hundred and forty-four patients with PDCA who underwent pancreatic resection were enrolled in this study: the maximum standardized uptake value (SUVmax) was able to identify two populations with a different prognosis. The patients with a lower SUVmax (≤3.65) had a significantly better survival rate than those with a higher SUVmax (*p* < 0.001). The SUVmax was an independent predictor of survival on multivariate analysis; therefore, it had the potential use in patients’ therapeutic management (i.e., the selection of patients for neoadjuvant therapy before surgery).

Another emerging and important factor impacting the outcome of oncologic patients is the cancer cachexia. In recent years, there has been a growing interest in the possible role of sarcopenia in influencing the morbidity and mortality of patients undergoing pancreatectomy for pancreatic adenocarcinoma [16,17]. In fact, among solid tumors, pancreatic cancer carries the highest prevalence of cancer cachexia and involuntary weight loss [18]. Pierobon et al. [14] from the University of Padua reviewed the literature and performed a meta-analysis comparing the outcomes of patients, with or without low muscle mass, undergoing pancreatic surgery for PDCA. These authors found that patients with a low muscle mass experienced a reduced OS (*p* < 0.001). However, the meta-analysis did not demonstrate the influence of low muscle mass on postoperative outcomes.

Most pancreatic cancer recurs after a tumor resection. Despite the fact that surgery for recurrent cancer is not recommended and rarely feasible, a series of patients with recurrent pancreatic cancer who underwent re-resection were increasingly reported in the English literature. Choi et al. [15] from Korea, reviewed the literature to assess the role of repeated pancreatectomy for patients with recurrent pancreatic cancer in the remnant pancreas. The median overall survival was 60 months after repeated pancreatectomy for isolated local recurrence. Although surgery cannot replace adjuvant chemotherapy, repeated pancreatectomy has a potential role in selected local recurrent PDAC.

Two studies [3,8] focused on the pancreatic fistula rate after pancreatic resection. Park et al. [8] evaluated the use of a flowable hemostatic matrix in preventing postoperative pancreatic fistula (POPF). Fifty-three patients were enrolled in a randomized trial, and the use of the flowable hemostatic matrix was found to be an independent negative risk factor for POPF at multivariate logistic regression analysis, particularly after distal pancreatectomy. In the study of Marino et al. [3], the incidence of POPF was evaluated in a minimally invasive pancreatectomy performed with robotic approach, an emerging surgical technique which has gained increased interest worldwide. After pancreatic resection, the pancreatic stump was anastomosed to the jejunum (*n* = 40) (PJ) or to the stomach (*n* = 20) (PG). The rate of clinically relevant POPF (12.5% vs. 10%, *p* = 0.82) did not differ between the two groups. Patients with PJ experienced more frequently intra-abdominal collections (7.5% vs. 0%, *p* = 0.002), but in this group there was a lower rate of post-pancreatectomy hemorrhage (2.5% vs. 10%, *p* = 0.003). On the contrary, patients with PG experienced a lower rate of POPF (33.3% vs. 50%, *p* = 0.003) in the high-risk group of patients.

Over the years, a growing number of patients were diagnosed with IPMNs, most likely as a consequence of a more extensive use of cross-sectional imaging. However, the management of this clinical entity remains controversial. Different clinical and radiological variables have been proposed in order to stratify the risk of the malignant degeneration of pancreatic IPMNs, and thus to guide their management. International consensus guidelines (ICG) recommend pancreatic resection for IPMNs with one or more “high-risk stigmata” (HRS), while patients with “worrisome features” (WF) should undergo a further assessment with endoscopic ultrasonography [19]. However, there is still a lack of accuracy in detecting the early invasive carcinoma in IPMNs. Serafini et al. [4] from the University of Padua (Italy) investigated the role of some systemic, inflammatory biomarkers in the diagnosis and prognosis of malignant, intraductal, papillary, mucinous neoplasms (IPMNs) of the pancreas. In 83 patients with histologically proven IPMN, the ratio of the C-reactive protein to the albumin ratio (CAR) was an independent predictor of high- grade dysplasia or invasive carcinoma in a multivariate analysis, and therefore, it could be useful to stratify the patients’ prognosis.

Two studies from the same institution focused on non-ductal neoplasms. Milanetto et al. [12] reported a series of seven patients with rare neuroendocrine tumors of the pancreas: the serotonin-secreting tumors. Six out of seven patients presented high urinary 5-HIAA and two patients presented with a carcinoid syndrome. In all cases, liver metastases were present at diagnosis and none of the patients underwent resection, but after a multimodality treatment (chemotherapy, somatostatin analogues and/or loco-regional liver ablation) a 5-year survival rate of 42.9% was achieved.

The same author [7] conducted an Italian, retrospective, multicentric study concerning the treatment of the pancreatic metastases of renal cell cancer (RCC-PMs), the most frequent secondary tumors of the pancreas. The authors considered the clinical-pathological characteristics, the therapeutic management and the DFS/OS, and they discussed the potential indications of pancreatic resection. They concluded that surgery could be considered for radically resectable RCC-PMs; both single and multiple PMs. There were no differences in disease recurrence when comparing limited pancreatic resections to standard pancreatectomies. A splenectomy and lymph node surgery could be avoided, since lymph node metastases were uncommon. In experienced hands, surgical resection was safe and effective, with more than one third of cases showing no disease recurrence after a median follow-up longer than 12 years. New studies are needed in order to establish how to combine the newly available target therapies with the surgical resection.

In conclusion, this Special Issue brings new insights on the outcomes and potential problems connected to multimodality therapy for pancreatic adenocarcinoma, potential prognostic factors influencing both surgery and chemotherapy, and novel strategies for the individualized treatment of patients with pancreatic neoplasms.

## Data Availability

Not applicable.
